# Supplementary material for: Early life exposures associated with risk of small intestinal neuroendocrine tumors
Source: PLoS One. 2020 Apr 23;15(4):e0231991. doi: 10.1371/journal.pone.0231991 (PMC7179894; doi:10.1371/journal.pone.0231991)
Supplement: S1 Table — (DOCX) [file pone.0231991.s001.docx]

Supplemental table 1. Sources of data used to characterize environmental and economic conditions of birth locales

| **Topic** | **Data Source and reference** | **Data elements** |
| --- | --- | --- |
| Historic and contemporary mining | Utah Geological Survey, Utah Mineral Occurrence System (<https://geology.utah.gov/map-pub/maps/interactive-maps/utah-mineral-occurrence-system/>)  Butler BS, Loughlin GF, Heikes VC. Ore deposits of Utah. Washington: Govt. Print. Off.; 1920.  Free Mining Claim Maps And Reports [Internet]. The Diggings™. [cited 2019]. Available from: <https://thediggings.com/>  History US. Utah Historical Quarterly, Volume 57, Number 4, 1989 [Internet]. Issuu. [cited 2019]. Available from: https://issuu.com/utah10/docs/uhq_volume57_1989_number4  Johnson M. Placer gold: deposits of Utah. Place of publication not identified: Gem Guides Book Co; 1995.  Lupton CT. The Deep Creek District of the Vernal Coal Field, Uinta County, Utah. Washington: G.P.O.; 1910  Palacios, Patsy; Luecke, Chris; and Robinson, Justin (2007) "Mineral activities within Rich County, Utah," *Natural Resources and Environmental Issues*: Vol. 14 , Article 10.  Available at: <https://digitalcommons.usu.edu/nrei/vol14/iss1/10>.  Mineral Resources [Internet]. Utah Geological Survey. [cited 2019]. Available from: https://geology.utah.gov/resources/mineral-resources/  Recent Articles [Internet]. Mindat.org - Mines, Minerals and More. [cited 2019]. Available from: https://www.mindat.org/  Welcome to Western Mining History [Internet]. Western Mining History. [cited 2019]. Available from: <https://westernmininghistory.com/>  Butler BS. Geology and ore deposits of the San Francisco and adjacent districts Utah. Washington; 1913.  Hughes CE. The Development of the Smelting Industry in the Central Salt Lake Valley Communities of Midvale, Murray, and Sandy Prior to 1900. 1990.  Lindgren W, Loughlin GF, Heikes VC. Geology and ore deposits of the Tintic Mining District, Utah, Issues 107-108. Washington: G.P.O.; 1919.  Murphy JR. The mineral resources of the territory of Utah: with mining statistics and maps. London: Trübner; 1872.  Raymond RW. Statistics of mines and mining in the states and territories west of the Rocky Mountains. Washington: Govt. Print. Off.; 1870.  Record of Decision Davenport and Flagstaff Smelters Site Operable Unit 1, Sandy, Utah. U.S. Environmental Protection Agency; 2002.  Strack D. Welcome To UtahRails.net [Internet]. UtahRails.net Home Page. 2019 [cited 2019]. Available from: https://utahrails.net/index.php  Whitley C. From the Ground Up A History of Mining in Utah. Logan, UT: Utah State University Press; 2006. | Name(s), location, commodities and production level, discovery date, abandonment date, workforce, supporting communities (variable information depending on mine and source) |
| Smelting | Same sources as for Heavy Industry and Mining |  |
| Heavy Industry | Salt Lake City Planning: Master Plans 2018 [updated 03/22/3028]. Available from: https://www.slc.gov/planning/2018/03/22/neighborhood-plans/.  Salt Lake City GIS Open Data. 2018.  USEPA. United States Environmental Protection Agency: Superfund Sites in Region 8 2018 [cited 2018]. Available from: <https://www.epa.gov/region8/superfund-sites-region-8>.  USEPA. Toxic Release Inventory Basic Plus Data Files 1987 - 2018. [cited 2018] Available at: <https://www.epa.gov/toxics-release-inventory-tri-program/tri-basic-plus-data-files-calendar-years-1987-2018>  Utah State Historical Society and various authors. (1995-1999). Utah Centennial County History Series, 29 volumes. <https://issuu.com/utah10/stacks/7656548957474267a733cb3bed39e1cb> |  |
| Agricultural activity | Arrington LJ. Beet sugar in the West: a history of the Utah-Idaho Sugar Company, 1891-1966. Seattle: Univ. of Washington; 1966.  Jeppeson S. Hyde Park, Utah History. Available from: http://hydepark.utahlinks.org/history.pdf  Midgley TK. Early exploration and settlement of the Tooele area, Utah. Provo: Brigham Young University; 1953.  Peterson CS. The "Americanization" of Utah's agriculture. The "Americanization" of Utah's agriculture. 1974;  Peterson CS. "Touch of the mountain sod" : how land united and divided Utahns, 1847-1985. Weber State College Press; 1989.  Powell A. Agriculture in Utah [Internet]. Utah History Encyclopedia. Available from: https://www.uen.org/utah_history_encyclopedia/a/Agriculture.shtml  Raty LS. A history of Wasatch county, 1859-1899. 1954.  Utah History Encyclopedia. [cited 2018]. Available from: <https://www.uen.org/utah_history_encyclopedia/> | Commodity, irrigation, time period, location, nearby communities |
| Settlement size, rurality Connectedness Commerce | Powell AK. Population. Found in Utah History Encyclopedia. Population by Communities and Census Precincts From 1890 to 1990. [cited 2018]. Available from: <https://www.uen.org/utah_history_encyclopedia/>p/POPULATION.shtml  Utah State Historical Society and various authors. (1995-1999). Utah Centennial County History Series, 29 volumes. <https://issuu.com/utah10/stacks/7656548957474267a733cb3bed39e1cb>  VanCott JW. Utah Place Names. Salt Lake City: University of Utah Press; 1990 | Location of rail service |
| Shipping/transport | See entries for Heavy Industry, Mining and Agriculture |  |
|  |  |  |
