# Supplementary material for: Early life exposures associated with risk of small intestinal neuroendocrine tumors
Source: PLoS One. 2020 Apr 23;15(4):e0231991. doi: 10.1371/journal.pone.0231991 (PMC7179894; doi:10.1371/journal.pone.0231991)
Supplement: Attachment — Submitted filename: Response to reviewers.docx [file pone.0231991.s003.docx]

Thank you to the reviewers for their time expertise and suggested changes. Below, we respond to each point. In addition to these changes, we clarified wording throughout. In the process we also noted that the last case in our data set was born in 1982. Some of the analysis indicated 1985, but this was incorrect, it is 1982. The results are not changed because all cases are matched to controls based on birth years and exposures are assigned based on birth year.

Reviewer 1:

1. I would remove the non-significant OR from the abstract and more simply indicate that other exposures approach significance. While the 0.05 is an arbitrary level, it is the standard convention.

This has been removed.

2. Line 50 - Perhaps rearrange to say "...epidemiological studies have been limited." (trivial I know)

This has been changed as suggested.

3. Line 57 - Indicate the countries included in the meta-analysis. The rest of the manuscript is based on Utah exposure, which might be different than other places.

We now indicate sources of meta-analysis: U.S.A. and Europe.

4. Lines 60-61 - Re-word the sentence so its clear that a large proportion of residents hold religious proscriptions. As written it almost comes across as promulgated by the state of Utah.

Good point! We have reworded this. “the majority of the population being members of the Church of Jesus Christ of Latter-Day Saints (a.k.a. Mormon) which has proscriptions against drinking and smoking…”

5. Line 72 - While you define eligible life events later, please provide a few examples here ("...with a life event (e.g. birth, marriage, etc.)...").

Thank you for this suggestion; we have now provided examples (e.g. birth, marriage, death, driver license renewal or hospitalization).

6. Line 89 - What proportion of the Utah population is part of the >11 people?

We have clarified this description to now read “UPDB is a shared resource located at the University of Utah and consists of computerized data records for >11 million deceased or living people dating from the 1700’s to the present. (<http://healthcare.utah.edu/huntsmancancerinstitute/research/updb/>). Although the population is dynamic, more than 95% of current adult residents in Utah (2.3 million) are represented in UPDB.”
7. Line 96 - Does "..this year." mean 2019?

This has been clarified to be 1966.

8. Line 105 - Seems close to half of cases are excluded. What types of bias might be represented here (e.g. lower income more likely to remain in state)?

We realize the original wording was misleading to both reviewers. Nearly half were not born in Utah which was our sole exclusion. When looking at an older population in Utah, such as those diagnosed with cancer, it is very typical to have approximately half born out of the state. Because we matched our controls to be individuals who were similarly born in Utah, the same year, we do not feel we have introduced biases. We have reworded it: “Cases were identified from the UCR with SINT diagnosis (ICDO codes C170-C173 or C178-C179 and a histology code of 8240 or 8241) from 1948 to 2014 (n=745). Three hundred and ninety cases fulfilled the inclusion criteria of individuals born in Utah.”

9. Lines 110-113 - Couple things
9a - Make clear you have alcohol/tobacco data for each/all cases and controls.
9b - How are/were multiple records resolved (e.g. an individual might have 10 entries for alcohol use over 20 years, ranging from 0 consumption to 3 drinks/day)?
9c - What type(s) of bias and possible error might be associated with lack of data prior to 1996? Some people have died before this time.

We have revised this section with more description of the process. Because cases and controls are matched based on birth year and follow up time in UPDB, we should be returning matched data sources for cases and controls. 9a) We now include the percent of cases and controls that have medical records in UPDB for 1996 or later (86%). 9b) We used any positive indication in the medical record, so if there were multiple reports and one was a positive indication, this individual was assigned “yes, indicated”. This is more clearly described in this section now. 9c) For cases and controls who died prior to 1996, contributing cause of death from tobacco or alcohol noted on a Utah death certificate was used to provide an indication of use. This is now included in description.

10. Line 164 - How were social conditions and economic activities for each locale determined and consolidated? Assessed for each year, averaged over a time period, other? Seems a factory might have a large economic splash for 10 years in a period, but not the rest before opening/after closing.

We have expanded our description of these methods. Generally we consulted histories covering an area and conducted more in depth research where information was lacking. Fortunately there are several comprehensive resources that we relied on. One of the most valuable sources is a 29 volume historical series compiled between 1995-1999. Each volume covers one of the 29 Utah counties and systematically covers major communities, including their economic and social conditions from settlement to the 1980s. Each volume runs 300 to 400 pages. This resource is reference #39, Bradley, et al.

11. Table 1 - better define how rural/urban were determined.

Thank you for pointing this out. City was designed to capture whether the subject was born in a significant economic or political settlement of the area for the time period of the birth. This designation varied over time and some locales that were important regional economic centers lost that role as transportation improved and/or the major economic activity of the area diminished. Rural was designed to capture areas that were not in proximity to the major metropolitan centers. This also could vary over time as improvements in transportation and growth effectively connected locales that were effectively quite isolated during earlier time periods. Locales not defined as City or Rural are small towns that are not contiguous with metropolitan areas but not isolated from a urban area. We have revised the descriptions in Table 1 to better describe the constructs.

12. Lines 179-181 - The justification/definitions for the 3 time periods could be strengthened, and maybe mover earlier in Methods. Up to this point it almost seems like year by year modeling.

As noted above we made substantial changes to better explain the characterization of the birth cities. We defined time intervals for each industrial activity and then linked each case/control to these factors based on birth locale and year. The three periods were subsequently used to estimate separate models for each Period (stratified analysis). As the periods were relevant to the modeling, we have described the Periods in that section.

13. Line 201 - Indicate "...and three ADDITIONAL cases."

This has been fixed, both here and the next line to indicate that they are additional cases.

Reviewer #2: This is a well written manuscript that presents original research investigating potential environmental risk factors for small intestinal neuroendocrine tumors (SINT). SINT are rare but have been increasing in incidence over the past 40 years and little is known about the etiology. The authors conducted a record linkage case-control study using the Utah Population Database (UTPD), which contains residential histories and life event data. Using a spatial scan statistic, they identified a cluster based on birth residence in an area that historically had coal mines. Using residence location at birth they identified increased risk for SINT among those living near construction and mining operations before 1940. Lower risk was observed in rural areas among those born in a later period (1945-1985). The authors went to considerable effort to link birth residences in their study to historical locations of mines, construction sites, and agriculture and this appears to be the first effort to link these historical data to cancer incidence data in Utah. This exploratory analysis of SINT incidence in relation to characteristics of the birth residence makes an important contribution to the limited literature on environmental exposures and these cancers. My main comments are about providing more detail on the both the historical environmental database and the UTPD, which are important for understanding and interpreting their results.

Major comments
1. I would like to see more details about the exposure database, which was reconstructed over an approximately 100-year period. Please describe how accurately you were able to locate the industries and agricultural areas and the completeness of your data sources over time and by industry type. What were your sources of the agricultural data back through time? Were USGS land cover maps used for the most recent period? A supplemental table documenting some of these details would be helpful for understanding the data sources and potential gaps or uncertainties in these spatial data.

Thank you for pointing out the aspects of our study methods that were not clear. Birth locale is the geographic unit of analysis, so we were assigning the environmental and economic characteristics to that birth locale. We have added a supplemental table that documents the major sources of information we used. Thank you for this suggestion.

We are confident that the general historical references captured all major industrial facilities, manufacturing, mining and other large construction/public works projects. Local planning documents for the Salt Lake City metropolitan area also provided specific business information. The historical documents also discussed the specific types of agricultural activity as well as the general time periods for the development of irrigation.

2. How complete are the residential history data in the UTPD? Please describe this for the study population and briefly describe factors associated with completeness (e.g., gender, birth year, etc). Although the authors reference several publications that use the UTPD, I think it’s appropriate to include more details about the database including the relative availability of residential data sources back through time. Please clarify if a LexisNexis search was used to obtain some of the historical residence information and if not, why wasn’t this done?

Clearly we did not adequately describe the methods used to establish the subjects’ residential location. We have clarified that the study focuses on conditions around the time of the subject’s birth. As described in the revised methods, we were able to use the mother’s residential address from birth certificates to establish birth locale for 96% of subjects, and either the birth city (location of hospital) or other address information for the remaining. As such we had birth locale for all cases and controls and did not need other sources of address.

3. Little is known about risk factors for SINT, particularly whether early life is a critical time period for exposures or if long-term exposures are most important. The authors mention that the use of one residence location to characterize exposure is a limitation of their study. They also point out the importance of evaluating cumulative exposures in future studies, with which I agree. No doubt this would be challenging to do for SINT since it may substantially increase the geographic extent of their exposure assessment. However, given that at least some residential history data are available for their cases and controls, I would like to see information on the percentage of the residence histories represented by the birth residence. Also, a sensitivity analysis limited to those with longer duration at this address would be informative.

As now noted in the Discussion, birth certificates account for only 11% of all addresses available in the UPDB, but are one of the more important sources of address for earlier time periods, accounting for approximately 45% of available addresses for the study period. Determining the length of residence at the birth locale would require substantial effort creating residential histories and is beyond the scope of this study. This is a strong interest of ours and we are pursuing this idea.

4. Related to comment #3, the authors mention in the discussion that an evaluation of cumulative exposures would be particularly challenging for rarer cancers due to power issues. Is this because many cases would be excluded due to a lack of substantial residence histories? Please clarify.

Yes, as any gap in a residential history would lead to incomplete exposure characterization. We have clarified this point in the manuscript.

5. The introduction mentions increasing incidence of colorectal cancer in younger adults and the hypothesis that this increase is related to early life exposures. Did you conduct analyses separately for younger and older cases of SINT? It would be interesting to know if risk differed by age at diagnosis. Further, were there any differences by gender? Higher risk among men would suggest that occupational exposures might be important.

We did not conduct analysis separately for the younger cases because the numbers were small (66 cases). This would be a valuable exploration in a larger study.

We have added to the introduction “Similar to the increasing incidence of colorectal cancer in younger adults, SEER data (1975-2016) shows a 3.4 annual percent increase in SINT incidence under age 50y (SEER*Stat 8.3.6). However, diagnoses of SINT over age 50y are also increasing (2.4 annual percent increase) while colorectal cancers in this age group have been decreasing.

Men and women do not significantly differ in either age group.

6. Cases were from 1948 through 2014. However, cancer data is incomplete before 1966 when the tumor registry was established. How confident are you that the cases prior to 1966 are representative of all SINT cases in this earlier period? Can you conduct sensitivity analyses excluding cases and their matched controls prior to 1966?

There were only 8 cases prior to 1966. When we examined their residence and demographics, they were similar to the other 382 cases. Because the numbers are so small, a sensitivity analysis would be uninformative.

In the text, we now include “Three hundred and ninety cases fulfilled the inclusion criteria of individuals born in Utah. Eight were diagnosed before 1966 (2%). There was no geographical clustering of these 8 early cases, they represented 6 counties both urban and rural and were similar in demographics to the cases diagnosed after 1966.”

Specific Comments:
Abstract
1. include the years of case ascertainment and the age range of the cases. Conclusion sentence seems too strong given the exploratory nature of this analysis.

Years and age are now included. Conclusion sentence was softened to include “may”.

Introduction
2. Tobacco use is listed as one of the factors that may contribute to the rise in SINT; however, tobacco use has gone down over time. Please clarify this point.

We have added this sentence to point out the trends in tobacco use and explain its possible influence “Although tobacco use has declined since its peak in the 1960’s, the lag time from tobacco exposure to cancer later in life is consistent with the recent rise in incidence of SINT [7].” If one considers someone born in 1940, smoked in their 20’s and 30s, diagnosed in 2000, their diagnosis would fall into the range of rising incidence.

Methods
3. Page 5. Case and control selection. Almost ½ of the 745 cases identified in the Utah Cancer Registry did not meet the inclusion criteria. Please clarify the reasons for exclusion. Provide information on the percent of cases and controls for whom hospital location was used as a proxy for birth residence.

Reviewer 1 was also concerned about this and we respond: We realize the original wording was misleading to both reviewers. Nearly half were not born in Utah which was our sole exclusion. When looking at an older population in Utah, such as those diagnosed with cancer, it is very typical to have approximately half born out of the state. Because we matched our controls to be individuals who were similarly born in Utah, the same year, we do not feel we have introduced biases. We have reworded it: “Cases were identified from the UCR with SINT diagnosis (ICDO codes C170-C173 or C178-C179 and a histology code of 8240 or 8241) from 1948 to 2014 (n=745). Three hundred and ninety cases fulfilled inclusion criteria of individuals born in Utah, 8 were diagnosed before 1966 (2%).”

4. Using the UTM data that is available on some cases and controls, can you describe the positional errors in the locale centroids? Does this differ for urban and rural areas? Please comment on how these errors might result in exposure misclassification.

We have cleaned up the language throughout the manuscript to clarify that cases and controls were geocoded to their birth city (locale) and not to their residential address. Full residential addresses were not used to assign birth locales or UTMs (other than for a small proportion of SLC cases and controls whose addresses were used to aggregate individuals to historic neighborhoods). Given that each birth locale only corresponds to one set of UTM coordinates, and all cases and controls geocoded to a given birth locale have the same set of UTM coordinates, we do not have the spatial resolution to describe positional errors in the birth locale. In addition, we have clarified in the method section that the exposure categories for birth locales is not based on spatial relationships between the 16 exposure categories and the UTM location of the birth locale. Rather, the historical profiles for each of the 405 birth locales was constructed from multiple historical documents.

5. Since each birth locale can have multiple industries during the same time period (page 8), is it feasible to compute a metric that accounts for multiple industries? How common was a multiple industry exposure scenario?

In most cases, except in some parts of Salt Lake City, there was only a single major heavy industrial facility at any one point in time. There were cases where a birth locale hosted both a heavy industry and mining (1.8%), hard rock mining (2.4%), smelters (3.0%), and large construction projects (2.1%). Overall 1600 of the 4100 subjects (41%) were from a birth locale hosting one or more of these activities. While this composite variable could be used as an exposure variable, interpretation would be difficult as ‘exposure’ would have different meanings for different subjects.

Discussion
6. The large amount of missing data for smoking and alcohol intake should be acknowledged as a limitation. How was 'not indicated' information handled in the adjusted analysis? Are the percentages of smoking and alcohol intake in controls comparable to the Utah population in this time period/age range?

We have added to the discussion, "Although exposure analysis was adjusted for tobacco use, data on tobacco use prior to 1996 was limited to contributing cause of death information recorded on a Utah death certificate. Thus, it is likely that for some subjects, indication of tobacco use was misclassified, particularly for case and control subjects who died before 1996 in which exposure data were limited.” Although tobacco use in this study is higher that what has been historically reported for Utah (14% in 1998), rates are similar between cases and controls. The higher rate may be attributed to the difference between reporting of “ever used tobacco” (which is the case here) and “currently use tobacco”.

7. Did the authors consider how radioactive fallout from nuclear tests in the 1940s to 1960s might have exposed their study population?

This is an important question that we did consider but could not address. Dispersion maps show a significant predictive radiation dosage across all of Utah (https://en.wikipedia.org/wiki/Nevada_Test_Site). Because all of our cases and controls would have been similarly exposed over this time period, we were unable to test for this. We also do not have information on food-related exposures.

Sincerely

Deborah W. Neklason, PhD

Associate Professor, Research

Division of Epidemiology, Department of Internal Medicine

Huntsman Cancer Institute at University of Utah

[Deb.neklason@hci.utah.edu](mailto:Deb.neklason@hci.utah.edu)

(801)587-9882 office
